# Outcomes of Ischemic Stroke and Associated Factors Among Elderly Patients With Large-Artery Atherosclerosis: A Hospital-Based Follow-Up Study in China

**DOI:** 10.3389/fneur.2021.642426

**Published:** 2021-04-23

**Authors:** Qianqian Wu, Jingjing Cui, Yuanli Xie, Min Wang, Huifang Zhang, Xiaofei Hu, Fenghua Jiang

**Affiliations:** ^1^Department of Neurology, Dongying People's Hospital, Dongying, China; ^2^Department of Rehabilitation Medicine, Dongying People's Hospital, Dongying, China

**Keywords:** outcomes, ischemic stroke, risk factors, elderly, large-artery atherosclerosis

## Abstract

Large-artery atherosclerotic (LAA) stroke is the most common subtype of ischemic stroke. However, risk factors for long-term outcomes of LAA stroke in the elderly Chinese population have not been well-described. Therefore, we aimed to assess outcomes and risk factors at 3, 12, and 36 months after LAA stroke onset among stroke patients aged 60 years and older. All consecutive LAA patients aged ≥ 60 years were prospectively recruited from Dongying People's Hospital between January 2016 and December 2018. The clinical features and outcome data at 3, 12, and 36 months after stroke were collected. Differences in outcomes and relationship between outcomes and risk factors were assessed. A total of 1,772 patients were included in our study (61.7% male, 38.3% female). The rates of mortality, recurrence, and dependency were 6.6, 12.6, and 12.6%, respectively, at 3 months after stroke onset. The corresponding rate rose rapidly at 36 months (23.2, 78.7, and 79.7%, respectively). We found the positive predictors associated outcomes at 3, 12, and 36 months after stroke onset. The relative risk (RR) with 95% confidential interval (CI) is 1.06 (1.02–1.10, *P* = 0.006) at 3 months, 1.06 (1.02–1.10, *P* = 0.003) at12 months, and 1.10 (1.05–1.15, *P* < 0.001) at 36 months after stroke onset for age; 1.09 (1.01–1.19, *P* = 0.029) at 12 months for fasting plasma glucose (FPG) level; 4.25 (2.14–8.43, *P* < 0.001) at 3 months, 4.95 (2.70–9.10, *P* < 0.001) at 12 months, and 4.82 (2.25–10.32, *P* < 0.001) at 36 months for moderate stroke; 7.56 (3.42–16.72, *P* < 0.001) at 3 months, 11.08 (5.26–23.34, *P* < 0.001) at 12 months, and 14.30 (4.85–42.11, *P* < 0.001) at 36 months for severe stroke, compared to mild stroke. Hypersensitive C-reactive protein (hs-CRP) level was an independent risk factor for mortality at different follow-up times, with the RR (95%) of 1.02 (1.01–1.02, *P* < 0.001) at 3 months, 1.01 (1.00–1.02, *P* = 0.002) at 12 months. White blood cell count (WBC) level was associated with both stroke recurrence (RR = 1.09, 95%CI: 1.01–1.18, *P* = 0.023) and dependency (RR = 1.10, 95%CI: 1.02–1.19, *P* = 0.018) at 3 months. In contrast, a higher level of low-density lipoprotein cholesterol (LDL-C) within the normal range was a protective factor for recurrence and dependency at shorter follow-up times, with the RR (95%) of 0.67 (0.51–0.89, *P* = 0.005) and 0.67 (0.50–0.88, *P* = 0.005), respectively. These findings suggest that it is necessary to control the risk factors of LAA to reduce the burden of LAA stroke. Especially, this study provides a new challenge to explore the possibility of lowering LDL-C level for improved stroke prognosis.

## Introduction

Stroke affects more than 10 million people worldwide annually and is the second-most common cause of death and the third-most common cause of long-term disability (1). Stroke can seriously affect the quality of life of patients and initiates a heavy burden on the families of patients and society (2). Large-artery atherosclerotic (LAA) stroke is the most common subtype of ischemic stroke, especially among Asian population accounting for about 33%, moreover, LAA increasing fastest among all subtypes reaching 5.7% annually (3). Compared with other stroke mechanisms, patients with macrovascular atherosclerosis such as internal carotid artery, intracranial vessel, or posterior circulation atherosclerosis had the highest risk of first stroke and early stroke recurrence (4, 5).

Underlying mechanisms of stroke caused by large atherosclerosis are diverse, including arterial-to-arterial emboli, *in situ* thromboembolism, hemodynamic impairment, and branching occlusive disease (BOD), and lesions could be in the lacunae, subcortical, cortical, or these bindings (6). Different studies on the prognosis and risk factors of ischemic stroke subtypes have shown that dyslipidemia, hypertension, diabetes, and obesity affect the prevalence of stroke and stroke-related mortality (7–9). Other traditional cardiovascular risk factors such as sex, race, smoking, alcohol consumption, risk-reducing medication, blood glucose level, and obesity can also increase the progression of atherosclerosis and thus affect the prognosis of this subtype of stroke (10). Furthermore, homocysteine and hs-CRP levels have also predicted stroke-related mortality (11, 12).

Although previous studies explored the prognostic outcomes of LAA stroke, few have explored the risk factors that affected the prognosis of this subtype of stroke among elderly patients. Therefore, we aimed to assess the outcomes and their risk factors at 3, 12, and 36 months after LAA stroke onset among stroke patients aged 60 years and older.

## Materials and Methods

### Participants

This was a prospective, single-center, hospital-based study that recruited patients from the stroke unit of Dongying People's Hospital, a tertiary general hospital in Shandong province, China, between January 2016 and December 2018, and all information of following-up was collected before January 2020. Consecutive patients who experienced a first LAA ischemic stroke were recruited. Patients who died before the imaging examination, those who experienced transient ischemic attack, and those under 60 years old were excluded.

The study was approved by the ethics committee for medical research at Dongying People's Hospital. Written informed consent was obtained from each participant during recruitment.

### LAA Diagnosis Criteria

We selected LAA stroke [the Trial Org 10172 in Acute Stroke Treatment (TOAST) classification] patients who were diagnosed by clinicians according to the following criteria (13): (a) clinical findings include those of cerebral cortical impairment or brain stem or cerebellar dysfunction; (b) cortical or cerebellar lesions and brainstem or subcortical hemispheric infarcts > 1.5 cm in diameter on CT or MRI; and (c) supportive evidence by duplex imaging of a stenosis of >50% of an appropriate intracranial or extracranial artery.

### Definitions of Clinical Features

Clinical features included stroke severity, stroke risk factors (hypertension, diabetes mellitus, atrial fibrillation, hyperlipidemia, obesity, current smoking, and drinking), neurological function scores [National Institutes of Health Stroke Scale (NIHSS), Barthel Index (BI), and modified Rankin Scale (mRS) scores], and laboratory test values [levels of fasting plasma glucose (FPG), total cholesterol (TC), triglycerides (TG), high-density lipoprotein cholesterol (HDL-C), low-density lipoprotein cholesterol (LDL-C), high-sensitivity hypersensitive C-reactive protein (hs-CRP), white blood cell count (WBC), and blood platelets count (BPC)].

Stroke severity was categorized into three groups according to the National Institutes of Health Stroke Scale (NIHSS) score: mild (NIHSS score: ≤7), moderate (NIHSS score: 8–16), and severe (NIHSS score: ≥17) (14). Hypertension was defined as systolic blood pressure ≥ 140 mmHg, diastolic blood pressure ≥ 90 mmHg, or taking medication for hypertension. Diabetes mellitus was defined as a FPG level ≥ 7.0 mmol/L or taking medication for diabetes. Obesity was defined as a body mass index of ≥ 28.0 kg/m^2^. Atrial fibrillation (AF) was defined as defined as a self-reported previous history of AF by patients or the presence of AF detected using 12-lead electrocardiography during hospitalization. Current smoking was defined as smoking ≥1 cigarette per day for more than 1 year and current drinking as drinking alcohol at least once per week for more than 1 year.

### Outcome Assessments

Outcomes included mortality, recurrence, and dependency at 3, 12, and 36 months after stroke onset. Mortality was defined as all-cause cumulative death during the follow-up period. Recurrence was defined as new-onset focal or global neurological dysfunction of vascular origin occurring >30 days after the initial stroke. Dependency was defined as an mRS score > 2.

Follow-up evaluations were conducted at 3, 12, and 36 months after stroke onset for all patients by the same trained senior neurologist in a face-to-face interview, except for patients who were re-examined in their local hospitals for whom information was collected by telephone. The investigator contacted the relatives or caregivers of survivors who could not be reached by telephone.

### Statistical Analysis

Continuous variables (age, FPG, TC, TG, hs-CRP, WBC, BPC, HDL-C, and LDL-C) are presented as means and standard deviation (SD). Categorical variables (stroke severity, hypertension, diabetes mellitus, obesity, current smoking, and current drinking) are presented as numbers with frequency. In the univariate analysis, continuous variables were compared with Student's *t*-tests and categorical variables were compared using chi-squared tests. In the multivariate analysis, dependent variables were stroke outcomes at 3, 12, and 36 months after stroke onset and the independent variable were variables which *P* ≤ 0.05 in the univariate analysis. The results of multivariate analysis were expressed as the adjusted relative risk (RR) and 95% confidence interval (CI). *P*-values ≤ 0.05 were considered statistically significant. SPSS for Windows (version 22.0; SPSS Inc., Chicago, IL, USA) was used for analyses.

## Results

### Baseline Characteristics of Patients With LAA

In this study, a total of 1,772 patients (≥60 years) with LAA ischemic stroke were involved in this follow-up study. The mean age was 70.60 years; 1,094 (61.7%) patients were men, and 678 (38.3%) were women. According to stroke severity, patients were classified into three grades: 63.2% mild, 27.1% moderate, and 9.7% severe. The prevalence of hypertension, AF, diabetes, and hyperlipidemia at baseline was 75.6, 6.9, 34.0, and 26.2%, respectively, smoking and drinking rates were 32.8 and 14.7%, respectively. The rate of obesity for these individuals was 13.4% at baseline. Average laboratory test values were FPG, 6.89 ± 2.92 mmol/L; TC, 4.92 ± 1.13 mmol/L; TG, 1.51 ± 1.01 mmol/L; HDL-C, 1.09 ± 0.30 mmol/L; LDL-C, 2.96 ± 0.88 mmol/L; and WBC, 7.78 ± 2.65 × 10^9^/L (Table 1).

**Table 1:** Baseline characteristics of patients with LAA stroke (age ≥ 60 years) stratified by sex group.

**Characteristics**	**Men**	**Women**	**Total**
Case, *n* (%)	1,094 (61.7)	678 (38.3)	1,772
**Stroke severity**, ***n*** **(%)**			
Mild	712 (65.1)	407 (60.0)	1,119 (63.2)
Moderate	290 (26.5)	190 (28.0)	480 (27.1)
Severe	91 (8.3)	81 (11.9)	172 (9.7)
**Clinical risk factors**, ***n*** **(%)**			
Hypertension	774 (70.7)	566 (83.5)	1,340 (75.6)
Diabetes mellitus	333 (30.4)	270 (39.8)	603 (34.0)
Atrial fibrillation	66 (6.0)	56 (8.3)	122 (6.9)
Hyperlipoidemia	286 (26.1)	178 (26.3)	464 (26.2)
Obesity	100 (9.1)	138 (20.4)	238 (13.4)
Current smoking	487 (44.5)	95 (14.0)	582 (32.8)
Current drinking	250 (22.9)	10 (1.5)	260 (14.7)
**Laboratory tests, mean (SD)**			
FPG, mmol/L	6.71 (2.84)	7.16 (3.02)	6.89 (2.92)
TC, mmol/L	4.65 (1.01)	5.38 (1.18)	4.92 (1.13)
TG, mmol/L	1.41 (0.89)	1.68 (1.17)	1.51 (1.01)
HDL-C, mmol/L	1.05 (0.30)	1.16 (0.29)	1.09 (0.30)
LDL-C, mmol/L	2.80 (0.80)	3.21 (0.95)	2.96 (0.88)
WBC,10^9^/L	7.86 (0.73)	7.63 (2.51)	7.78 (2.65)
BPC, 10^9^/L	208.44 (93.32)	234.73 (71.64)	218.43 (86.65)
Age, year, mean (SD)	70.08 (7.59)	71.44 (7.38)	70.60 (7.54)
**Neurological function, median (range)**			
NIHSS	5.00 (8)	6.00 (8)	6.00 (8)
BI	60.00 (50)	55.00 (45)	50.00 (50)
mRs	3.00 (2)	3.00 (2)	3.00 (2)

Of these patients, there were 93.5% (1,657/1,772), 88.3% (1,171/1,326), and 82.0% (341/416) patients finished the following-up at 3 months, 1 year, and 3 years after stroke, respectively.

### Outcomes at 3, 12, and 36 Months After Stroke Onset

The rates of mortality, recurrence, and dependency rose rapidly after stroke onset. At 3 months after onset, the rates of mortality, recurrence, and dependency were 6.6, 12.6, and 12.6%, respectively, rising to 10.8, 34.3, and 33.0% at 12 months and 23.2, 78.7, and 79.7% at 36 months (Table 2).

**Table 2:** Factors associated with outcomes at 3-, 12-, and 36-month after stroke among patients with LAA by univariate analysis.

**Characteristics**	**3 months**			**1 year**	**3 year**				
	**Mortality**	**Recurrence**	**Dependency**	**Mortality**	**Recurrence**	**Dependency**	**Mortality**	**Recurrence**	**Dependency**
Total, *n* (%)	110 (6.6)	196 (12.6)	195 (12.6)	127 (10.8)	371 (34.3)	345 (33.0)	79 (23.2)	236 (78.7)	208 (79.7)
**Sex**, ***n*** **(%)**									
Men	71 (6.9)	124 (13.0)	123 (12.9)	78 (10.9)	231 (35.2)	216 (34.0)	45 (22.1)	140 (79.1)	128 (80.5)
Women	39 (6.1)	72 (12.1)	72 (12.1)	49 (10.7)	140 (33.0)	129 (31.6)	34 (25.0)	96 (78.0)	80 (78.4)
**Stroke severity**, ***n*** **(%)**									
Mild	24 (2.2)^*^	109 (10.3)^*^	108 (10.2)^*^	27 (3.6)^*^	217 (29.6)^*^	211 (29.0)^*^	17 (8.5)^*^	146 (75.3)	139 (76.0)^*^
Moderate	45 (10.2)	68 (17.1)	68 (17.1)	53 (16.6)	121 (42.8)	111 (41.6)	39 (36.8)	74 (84.1)	58 (86.6)
Severe	41 (29.9)	18 (18.6)	18 (18.6)	47 (49.0)	32 (50.8)	22 (44.9)	23 (67.6)	16 (88.9)	11 (100)
**Clinical risk factors**, ***n*** **(%)**									
Hypertension	86 (6.9)	158 (13.6)	158 (13.6)^*^	101 (11.7)	284 (35.8)	263 (34.5)	60 (24.4)	168 (78.9)	150 (80.6)
Diabetes mellitus	33 (5.9)	62 (11.8)	62 (11.8)	45 (11.5)	124 (34.0)	111 (31.9)	30 (24.4)	89 (80.9)	78 (83.9)
Atrial fibrillation	14 (12.6)^*^	17 (17.3)	17 (17.3)	18 (22.5)^*^	31 (45.6)^*^	27 (43.5)	8 (36.4)	16 (84.2)	13 (92.9)
Obesity	17 (7.8)	21 (10.4)	21 (10.4)	18 (12.6)	50 (38.5)	46 (36.8)	14 (28.6)	38 (88.4)	31 (88.6)
Current smoking	25 (4.7)^*^	65 (12.7)	64 (12.5)	25 (7.1)^*^	109 (32.8)	105 (32.1)	15 (16.9)	61 (75.3)	55 (74.3)
Current drinking	8 (3.3)^*^	30 (12.8)	29 (12.3)	9 (5.2)^*^	50 (30.3)	49 (30.1)	6 (14.0)	33 (80.5)	29 (78.4)
**Laboratory tests, mean (SD)**									
FPG, mmol/L	7.91 (3.43)^*^	7.38 (3.57)^*^	7.39 (3.58)^*^	7.86 (3.42)^*^	6.83 (3.11)	6.79 (3.11)	7.26 (3.43)^*^	6.38 (2.26)	6.31 (2.02)
TC, mmol/L	4.96 (1.25)	4.91 (1.23)	4.91 (1.23)	4.99 (1.28)	4.89 (1.07)	4.87 (1.06)	5.10 (1.23)^*^	4.86 (1.01)	4.80 (1.01)
TG, mmol/L	1.28 (0.68)^*^	1.50 (0.84)	1.51 (0.84)	1.41 (1.02)	1.50 (1.03)	1.46 (0.96)	1.22 (0.59)	1.40 (0.78)	1.41 (0.80)
HDL-C, mmol/L	1.16 (0.56)	1.13 (0.28)^*^	1.14 (0.28)^*^	1.15 (0.52)	1.10 (0.28)	1.10 (0.29)	1.13 (0.31)^*^	1.03 (0.26)	1.02 (0.26)
LDL-C, mmol/L	3.09 (1.03)	2.83 (0.87)^*^	2.83 (0.87)^*^	3.10 (1.05)	2.99 (0.86)	2.99 (0.86)	3.28 (1.03)^*^	3.10 (0.84)	3.06 (0.84)
WBC,10^9^/L	9.70 (3.35)^*^	8.11 (3.47)^*^	8.12 (3.48)^*^	9.39 (3.36)^*^	7.96 (3.13)^*^	7.84 (3.06)^*^	9.09 (3.08)^*^	7.48 (2.97)	7.33 (2.97)
BPC, 10^9^/L	211.48 (80.32)	219.58 (78.01)	220.22 (77.71)	213.70 (88.84)	214.06 (72.61)	211.96 (68.05)	219.43 (82.40)	214.80 (69.76)	210.86 (65.82)
HCY, μmol/L	14.51 (8.54)	14.80 (10.18)	14.84 (10.21)	15.18 (9.38)	16.32 (12.83)	16.21 (12.89)	16.41 (13.69)	14.80 (11.02)	14.33 (9.64)
hs-CRP, mmol/L	40.07 (56.06)^*^	15.58 (29.47)^*^	15.70 (29.58)^*^	35.77 (55.15)^*^	13.64 (30.63)^*^	12.55 (27.43)^*^	25.70 (44.43)^*^	8.50 (20.44)	7.91 (19.29)
Age at onset, year, mean (SD)	74.32 (8.16)^*^	71.39 (8.12)^*^	71.44 (8.11)^*^	73.72 (7.87)^*^	71.17 (7.34)^*^	71.23 (7.42)^*^	73.32 (7.65)^*^	70.55 (6.75)	70.32 (6.70)

* *P < 0.05*.

### Univariate Factors Associated With Stroke-Related Outcomes

Table 2 shows that stroke severity, FPG level, hs-CRP level, WBC, and age were significantly correlated with 3-, 12-, and 36-month mortality (all *P* < 0.001 at 3- and 12-month; *P* < 0.001, *P* = 0.005, *P* < 0.001, *P* < 0.001, and *P* < 0.001 at 36-month). Smoking, drinking, and AF were associated with 3- and 12-month mortality (*P* = 0.025, *P* = 0.024, and *P* = 0.009 at 3-month; *P* = 0.007, *P* = 0.010, and *P* = 0.001 at 12-month). Moreover, TC, HDL-C, and LDL-C levels were correlated with 36-month mortality (*P* = 0.028, *P* = 0.004, and *P* = 0.050, respectively).

Stroke severity, hs-CRP level, WBC, and age were correlated with 3- and 12-month recurrence (*P* < 0.001, *P* = 0.005, *P* = 0.004, and *P* = 0.010 at 3-month; *P* < 0.001, *P* = 0.001, *P* = 0.002, and *P* < 0.001 at 12-month). FPG and HDL-C, and LDL-C were correlated with 3-month recurrence. Moreover, AF was associated with 12-month recurrence (*P* = 0.043). However, no risk factors were found associated with recurrence at 36 months after stroke onset.

Stroke severity was correlated with 3-, 12-, and 36-month dependence (all *P* < 0.001). Hypersensitive CRP level, WBC, and age were associated with 3- and 12-month dependence (*P* = 0.005, *P* = 0.003, and *P* = 0.007 at 3-month; *P* = 0.002, *P* = 0.018, and *P* < 0.001 at 12-month). Hypertension and FPG, HDL-C, and LDL-C levels were associated with 3-month dependence (*P* = 0.039, *P* = 0.004, *P* = 0.013, and *P* = 0.044, respectively).

### Predictors of Outcomes in the Multivariate Analysis

#### Mortality

**Figure 1:**
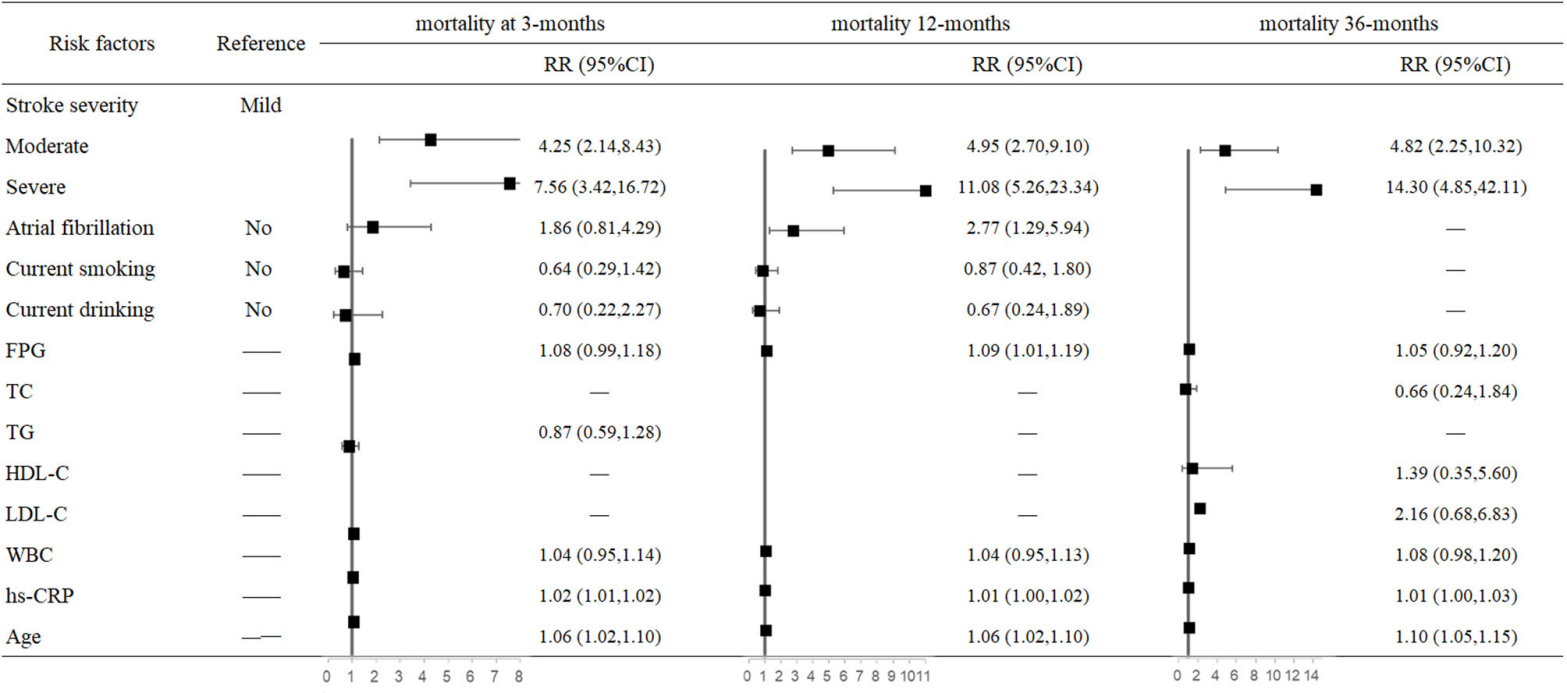
Predictors of mortality at 3, 12, and 36 months after stroke in the multivariate analysis.

In the multivariate analysis, both stroke severity and older age were associated with higher risk of mortality at 3, 12, and 36 months after stroke onset. Compared with mildly affected stroke patients, severely affected stroke increased mortality risk 6.56-fold (95% CI: 3.42–16.72, *P* < 0.001) at 3 months and 13.3-fold (95% CI: 4.85–42.11, *P* < 0.001) at 36 months after stroke onset. For each 1-year increased in age, mortality risk increased 6% at 3 months (95% CI: 1.02–1.10, *P* = 0.006) and 10% at 36 months after stroke onset. Moreover, AF increased 1.77-fold mortality risk (95% CI: 1.29–5.94, *P* = 0.009) only at 12 months. In addition, higher FPG (95% CI: 1.01–1.19, *P* = 0.029) and hs-CRP (95% CI: 1.00–1.02, *P* = 0.002) levels were also risk factors of mortality at 12 months after stroke onset (Figure 1).

#### Recurrence

**Figure 2:**
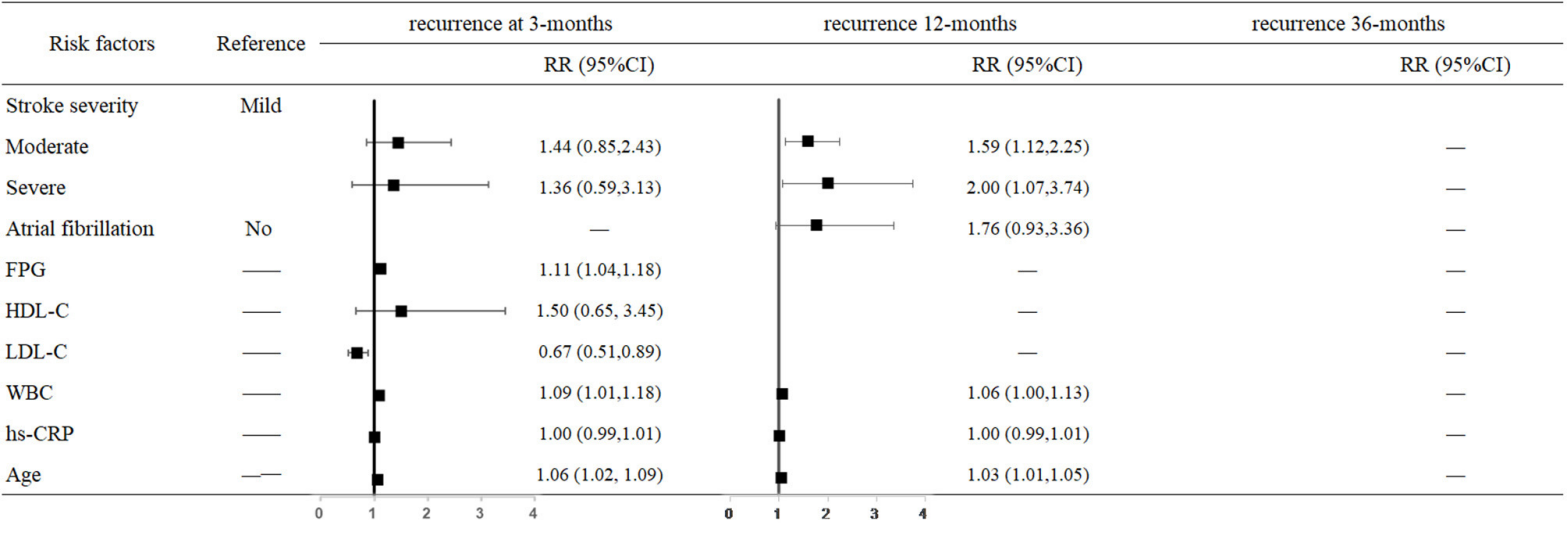
Predictors of recurrence at 3, 12, and 36 months after stroke in the multivariate analysis.

The hazard of mortality 3 months after stroke was associated with a higher FPG level (RR = 1.11, 95% CI: 1.04–1.18, *P* = 0.001), LDL-C level (RR = 0.67, 95% CI: 0.51–0.89, *P* = 0.005), WBC (RR = 1.09, 95% CI: 1.01–1.18, *P* = 0.023), and older age (RR = 1.06, 95% CI: 1.02–1.09, *P* = 0.001). The risk of 12-month recurrence in moderately affected and severely affected patients were 1.59-fold (95% CI: 1.12–2.25, *P* = 0.009) and 2-fold higher (95% CI: 1.07–3.74, *P* = 0.030) than in mildly affected patients. For each 1 × 10^9^/L increase in WBC, the risk of recurrence increased 6% (95% CI: 1.00–1.13, *P* = 0.050); for each 1-year increase in age, the risk of recurrence increased 3% (95% CI: 1.01–1.05, *P* = 0.005) (Figure 2).

#### Dependence

**Figure 3:**
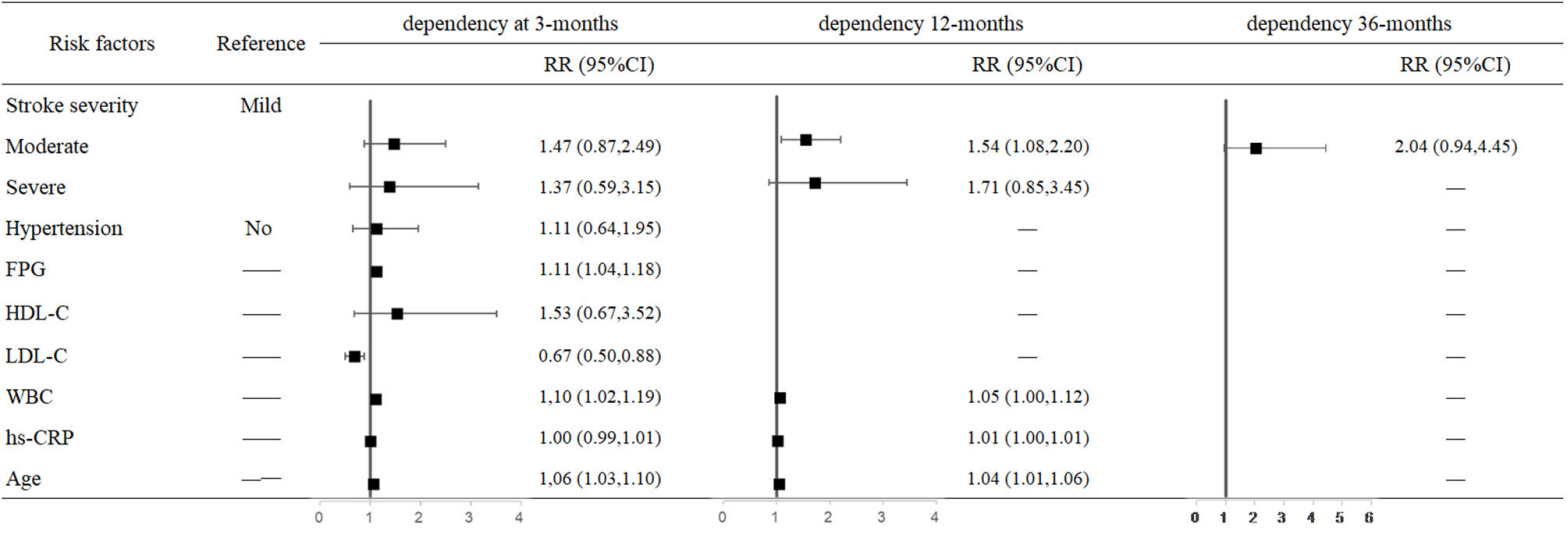
Predictors of dependency at 3, 12, and 36 months after stroke in the multivariate analysis.

For each unit increase in FPG and LDL-C levels and WBC, the risk of 3-month dependency increased 11% (95% CI: 1.04–1.18, *P* = 0.001), decreased 33% (95% CI: 0.50–0.88, *P* = 0.005), and increased 10% (95% CI: 1.02–1.19, *P* = 0.018), respectively. The risk of 12-month dependency in moderately affected patients was 54% higher than in mildly affected patients (95% CI: 1.08–2.20, *P* = 0.018). Moreover, older age increased the risk of dependency at both 3 months (RR = 1.06, 95% CI: 1.03–1.10, *P* < 0.001) and 12 months (RR = 1.04, 95% CI: 1.01–1.06, *P* = 0.002) after stroke onset (Figure 3).

## Discussion

This was a hospital-based follow-up study that explored outcomes and associated risk factors of LAA stroke in China in patients aged 60 years and older. The rates of mortality, recurrence, and dependency all dramatically increased between 3 and 36 months after stroke onset. Factors associated with this increase included older age, higher FPG levels, and stroke severity. Moreover, hs-CRP was an independent risk factor for mortality at 12 months after stroke onset. Furthermore, WBC level was associated with both stroke recurrence and dependency. In contrast, a higher LDL-C level was a protective factor for recurrence and dependency in short times.

Different cohort studies and population studies in Africa, such as TSIP, INTERSTROKE, and SIREN, have shown that older age was the strongest, immutable risk factor for stroke, and similar results have been reported for developed countries (15). Stroke involves cerebral vessel arteriosclerosis, which is a silent progressive process that can begin early in life and usually develops with age (16). Two hospital-based studies have shown that age was an important inverse predictor of functional recovery after ischemic stroke for 3 months, independent of stroke severity, characteristics, and complications, while the association of age with prognostic outcome is non-linear and extends to young stroke patients (17, 18). Other studies that examined risk factors for stroke subtypes based on the TOAST classification revealed that age was independently associated with short term (90-day) mortality in patients with LAA stroke (2, 19). The Atherosclerosis Risk in Communities (ARIC) study also reported that age was an independent risk factor for non-lacunar ischemic stroke subtypes after long term follow-up (20). Consistent with previous results, our study also clearly showed that older age increased the risk of outcomes at short and long term after the onset of LAA stroke, further validating the effect of age on the prognosis of this subtype of stroke.

Stroke severity was another important risk factor for stroke outcomes. One clinical trial included 1,281 stroke patients and used the TOAST classification to classify stroke and NIHSS to quantify neurological impairment at baseline. The results showed that NIHSS scores strongly predicted the likelihood of recovery in patients after stroke. Scores ≥16 indicated higher likelihood of death or severe disability, while score ≤ 6 indicated good recovery (21). A study in Italy also showed that NIHSS score was an important predictor of functional decline at 3 months (17). A study of gender differences in China also found that stroke severity was an independent risk factor for mortality, dependence, and recurrence in men and women at 3 and 12 months after stroke (22). In our study, the results showed an increased risk of prognosis (mortality, recurrence, and dependence) in moderate and severe relative to mild stroke over three periods. These results all confirmed that baseline NIHSS scores could be used as predictors of outcome after stroke.

Previous studies reported that high FPG level was correlated with poor outcomes of ischemic stroke (23–25). A study reported that acute ischemic stroke patients with a favorable neurologic outcome (90-day mRS scores ≤ 2) had a significantly lower baseline FPG level than those with an unfavorable neurologic outcome (6.6 ± 1.96 vs. 8.12 ± 4.02; *P* = 0.002) (24). The 6.5-year prospective Northern Manhattan Study (NOMAS) reported that diabetic subjects with elevated FPG level [HR 2.7 (95% CI 2.0–3.8)] were at increased risk of stroke, but those with a target FPG level (<7.0 mmol/L) were not, even after adjustment (26). Patients with type 2 diabetes were more likely to suffer from obesity, transient ischemic attack, and stroke caused by LAA and had a worse prognosis than in patients without diabetes (27). Moreover, increased FPG level on admission has been associated with poorer functional outcomes, irrespective of diabetes status after an acute ischemic stroke (28–30). Another study showed that the risk of ischemic stroke increased by 8.5% for each 1 mmol/L increase in FPG level, even in non-diabetic patients (23). The results of the present study showed that elevated FPG level was an independent risk factor for 3- and 12-month outcomes, further illustrating that high FPG level is a strong prediction factor for poor outcomes of LAA stroke; accordingly, early intervention and management should be considered to improve LAA stroke prognosis.

Increasing evidence suggests that inflammation is a key process in the pathogenesis of atherosclerosis. After acute stroke, CRP level is elevated due to tissue injury and infection, proportional to the severity of acute events. In addition, CRP level had the greatest increase in patients with LAA stroke, and baseline CRP level can independently predict the risk of stroke recurrence (31). Furthermore, serum hs-CRP level was associated with morphological characteristics of rapidly progressing carotid atherosclerosis, suggesting that hs-CRP level was a sensitive marker of the presence of active atherosclerotic disease (32). Furthermore, in a case-control study, serum CRP level in patients with all ischemic stroke subtypes were significantly higher in both the acute phase and at 3-month follow-up (33). Rost et al. found that high serum CRP level can predict the future risk of ischemic stroke in elderly patients (34). Another marker of inflammatory response is the leukocyte level. Compared with the quartile with lowest leukocyte counts at baseline, patients in the top quartile had higher risks for ischemic stroke (35). Evaluating WBC level was also related with both poor short- and long-term outcomes (36, 37). The association was strongest for those with LAA stroke compared with other subtypes (adjusted hazard ratio 1.48, 0.98–2.24) (38). The present results further suggest that CRP is an independent risk factors for mortality (both short- and long-term) and that WBC level is associated with both stroke recurrence and dependency, adding new evidence for the role of CRP and WBC level in stroke prognosis.

The relationships between LDL-C levels and stroke outcomes remain controversial. A study explored a population with higher LDL cholesterol that was >15 times more likely to have ischemic stroke than in a control group (39). Similarly, another showed that a LDL-C level of ≥2.6 mmol/L at admission was an independent risk factor for intracranial atherosclerotic stroke (9). In contrast, other studies reported no clear relationship between LDL-C and ischemic stroke (40, 41). Moreover, in contrast to the present results, a multi-center retrospective study revealed that compared with lower LDL-C levels, levels in the third quartile were less likely to exhibit unfavorable outcomes after ischemic stroke (42). Another national cohort study also supports this point, reporting that among the middle-aged and elderly Chinese population, a lower LDL-C level was associated with increased all-cause mortality risk (43). The current results suggest that LDL-C is a protective factor for short-term recurrence and dependence after stroke onset. Regardless, the average LDL-C level at baseline was classified as normal (<3.36 mmol/L) according to NCEP ATP III criteria (44). It may be that among patients with normal LDL-C levels, a higher level may be negatively associated with stroke outcomes: this hypothesis requires further prospective and well-designed studies for validation.

There are several limitations in the current study. First, the data in our study were collected from a single center in northern China, which may limit the generalization of our results. Moreover, patients recruited in our study were limited 60 years and older, comparisons between the present study and previous ones are limited by the age. Third, all the influencing factors were collected and measured at admission, and no follow-up information was available. Third, we were unable to analyze the impact of other risk factors such as medication adherence after discharge for prognosis. However, these factors have little impact on our current findings. Future studies need to refine information concerning changes in risk factors and drug administration during follow-up. Finally, we did not collect information about lipid lowering drugs, which may affect our evaluation on the association between LDL-C and outcomes.

## Conclusion

In conclusion, this was a hospital-based study that explored outcomes and associated risk factors among old Chinese LAA stroke patients. Older age, higher FPG level, and stroke severity were independent risk factors for mortality, recurrence, and dependence in LAA stroke. In addition, WBC was a risk factor for recurrence and dependency. Moreover, higher LDL-C level may reduce the risk of short-term recurrence and dependence following stroke in China. Therefore, it is necessary to control risk factors for LAA to reduce the burden of LAA stroke. In particular, this study provides a new challenge to determine whether lowering LDL-C level may improve stroke prognosis.

## Data Availability Statement

The raw data supporting the conclusions of this article will be made available by the authors, without undue reservation.

## Ethics Statement

The studies involving human participants were reviewed and approved by The Ethics Committee for medical research at Dongying People's Hospital. The patients/participants provided their written informed consent to participate in this study.

## Author Contributions

FJ contributed to the study design, performed data collection, data interpretation, critical review, and performed data analysis. QW and JC contributed to drafting of the article. QW, JC, YX, MW, HZ, and XH performed data collection, case diagnoses, and confirmation of case diagnoses. All authors read, revised, and approved the final version of the paper.

## Conflict of Interest

The authors declare that the research was conducted in the absence of any commercial or financial relationships that could be construed as a potential conflict of interest.
